# The impact of psychopathic traits on anxiety-related behaviors in a mixed reality environment

**DOI:** 10.1038/s41598-024-62438-9

**Published:** 2024-05-23

**Authors:** Alexander Voulgaris, Sarah V. Biedermann, Daniel Biedermann, Susanne Bründl, Lateefah Roth, Christian Wiessner, Peer Briken, Johannes Fuss

**Affiliations:** 1 Center for Psychosocial Medicine, Institute for Sex Research, Sexual Medicine, and Forensic Psychiatry, University Medical Center Hamburg-Eppendorf, Martinistr. 52, 20246 Hamburg, Germany; 2 Social and Emotional Neuroscience Group, Department of Psychiatry and Psychotherapy, Center of Psychosocial Medicine, University Medical Center Hamburg-Eppendorf, Hamburg, Germany; 3 Center for Translational Neuro- and Behavioral Sciences, Institute of Forensic Psychiatry and Sex Research, University of Duisburg-Essen, Alfredstr. 68-72, 45130 Essen, Germany; 4 Center for Experimental Medicine, Institute of Medical Biometry and Epidemiology, University Medical Center Hamburg-Eppendorf, Hamburg, Germany

**Keywords:** Human behaviour, Pathology

## Abstract

There is an ongoing debate about anxiety deficits in psychopathy and their possible impact on individual behavior. Data on actual anxiety- and threat-related behavior associated with psychopathy is still limited. We performed a mixed reality study using the elevated plus-maze (EPM) in a non-clinical sample (N = 160) to test anxiety-related behavior in relation to psychopathic personality traits measured through the Brief Questionnaire of Psychopathic Personality Traits (FPP). The psychopathy sum score correlated significantly with all measures of anxiety-related behavior on the EPM. Sensation seeking, but not general levels of acrophobia was moreover associated with psychopathic traits. Multivariate analyses revealed that the subscales *Fearlessness* and *Lack of Empathy* of the FPP predicted anxious behavior. Our findings are the first to demonstrate the relationship between psychopathic traits and actual behavior in an anxiety-inducing environment. This supports the low-anxiety hypothesis in psychopathy research. Implications for potentially harmful or risky behavior are discussed.

## Introduction

The personality construct of psychopathy consists of potentially severe deficits in behavior, emotion, and cognition, often categorized in the two broad dimensions of affective-interpersonal and antisocial-lifestyle, representing the two factors of Hare’s popular *Psychopathy Checklist-Revised*^1,2^. From a historical perspective, psychopathy was initially described as corresponding to a lack of anxiety or worry^3^. Early research indicated a significant degree of low anxiety and fearlessness as a core symptom of psychopathy, with Hervey Cleckley being the first to describe the item’s *lack of nervousness or anxiety*^3–6^. The first experimental research was conducted by David Lykken in 1957, describing findings of low physiological reactions to aversive stimuli in so-called “sociopaths”^7^, which led to the development of his “low-fear hypothesis” of psychopathy, with a notable impact on future research^8,9^ and conceptualizations of psychopathy.

Despite this early development, the existing research on psychopathy and anxiety remains mixed, with studies supporting this hypothesis^10–12^ and others reporting the contrary, questioning low anxiety as a critical feature of psychopathic personalities^13,14^. One reason for the mixed data on this topic of discussion may lie in the overlap between the constructs of anxiety and fear and the different methods of measuring psychopathy^15^. On this line, recent studies point to a more complex understanding of anxiety deficits in individuals with psychopathic traits. In their review and meta-analyses, Hoppenbrouwers et al.^16–18^ conclude that the term ’fear’ is often used generically in studies, stressing the relevance of precise definitions and differentiation between fear and anxiety. They further present evidence that psychopathic individuals rather have more context-specific deficits in threat detection and responsivity in behavioral and neurobiological measures^16^. In addition, other authors have noted that fear and anxiety are often used interchangeably in psychopathy research^15,19^.

With this in mind, it is essential to disentangle anxiety and fear for the following study. Anxiety can be described as hypervigilance in anticipation of a non-specific threat in the absence of a clear and acute danger^17^. In contrast, fear is an aversive unconditioned response to an immediate, specific threat stimulus often associated with one of the behavioral responses of fight, flight, or freeze^20–22^. In this vein, Öhman^23^, in his review focusing on fear and anxiety, proposed that fear occurs when a person is actively coping with a perceived threat, whereas anxiety occurs in a threatening situation without an effective means of coping. In her meta-analysis, Derefinko^19^cites findings that anxiety and fear are negligibly related to the total psychopathy scores and negatively correlated to Factor 1 scores (affective-interpersonal features such as superficial charm, grandiose sense of self-worth, lack of guilt, and callousness), while anxiety was positively related to Factor 2 scores (antisocial-lifestyle features such as need of stimulation, impulsivity, parasitic lifestyle, and irresponsibility). Further research has found that psychopathic individuals scoring high on affective-interpersonal items represent a group that exhibits anomalous response patterns associated with low anxiety and fearlessness^24–26^.

In neuroscience, experimental study designs identified a wide range of amygdala-mediated deficits implicated in the processing of fear and anxiety associated with psychopathy^27^. These have included abnormal affect-mediated acoustic startle modulation^28–30^ and evidence of impaired limbic-prefrontal circuitry^31^). In summary, existing data on psychopathy suggest specific cognitive deficits in emotionally-relevant information related to higher-order thinking rather than a general lack of anxiety or fear^15,32^. This is further supported by findings suggesting that, under specific circumstances, individuals high in psychopathy appear to process threat cues without any difficulty^33,34^.

The methods commonly used to assess anxiety, fear, or threat-related responses in psychopathy research vary widely. Most studies rely on self-report questionnaires, the ability to identify fear in others, autonomic measures, or learning to form aversive associations between neutral and threatening stimuli^16,35^. The existing literature focusing on specific anxiety- and threat-related behavior associated with psychopathy is limited. However, understanding this interplay is very important to individuals and society, as it may have implications for risky, potentially harmful, and antisocial behavior^36,37^.

The ‘gold standard’ for assessing anxiety-related approach-avoidance conflicts in animal research is the elevated plus-maze (EPM)^38,39^. It has been used in over 9500 published papers to date (Web of Science, 2023). Briefly described, mice or rats are placed in the middle of a four-armed elevated cross with two open and two closed arms, initially facing an open arm. For five minutes, duration and entries on the arms are measured by video tracking^40^. In this assay, an unconditioned approach-avoidance conflict is created, and anxiety is measured at the behavioral level by monitoring adaptive behavior without immediate threat, punishment, or reward^38^. The absence of an acute threat is relevant because the rodent fear test battery measures specific responses to predator approach or contact. In contrast, anxiety test battery measures for rodents, such as the EPM, assess risk assessment activity and inhibition of non-defensive behaviors in response to a potential threat^41^.

In a mixed reality study, Biedermann et al.^38,39,42^ translated the EPM from rodents to humans. They demonstrated the cross-species validity of open-arm avoidance as a translational measure of anxiety using the elevated plus-maze. In the study, individuals reported higher anxiety on open arms and avoided open-arm exploration, and individuals with higher subjective anxiety levels exhibited more avoidance of open arms. Notably, presence and immersion in the mixed reality setting were high, and face, content, and concurrent validity could be assumed, making this test a validated measure for assessing anxiety-related behavior in humans^43^.

### Aim of study

We conducted a mixed reality study using the EPM in a non-clinical sample to test anxiety-related behavior in relation to psychopathic personality traits. We hypothesized that a higher score on psychopathic traits would be associated with more approach and less avoidance on the EPM, which would lead to a higher expression of risky behavior and thus to more extended time on open arms, a higher number of entries to open arms and a lower latency for a first visit to open arms and exploration of the end of an open arm. Furthermore, we hypothesized that specifically the *Fearlessness* scale of the Brief Questionnaire of Psychopathic Personality Traits (FPP, 44), a brief self-report inventory based on the Psychopathic Personality Inventory, would correlate with the behavior mentioned above.

## Methods

### Participants and procedure

Healthy female and male volunteers (N = 170) were recruited through electronic and physical bulletin boards, mainly on the university campus and other public spaces. All subjects reported to be free of neurological or psychiatric disorders and were not regular users of recreational drugs or central nervous system medication. Volunteers gave written informed consent according to the Declaration of Helsinki. We obtained ethics approval from the Psychological Ethics Committee of the University Medical Center Hamburg-Eppendorf.

Ten subjects were removed from the analyses due to technical problems (n = 6) or not following the experimenters’ instructions (n = 4). This resulted in 160 participants. Before the behavioral testing phase, the participants were seated in a room in front of the behavioral laboratory. There, they were asked to fill out the following questionnaires: Sociodemographic variables, Sensation Seeking Scale V (SSSV)^44^, Acrophobia Questionnaire (AQ)^45^, and Brief Questionnaire of Psychopathic Personality Traits^46^. After completing the questionnaires, the participants were taken to the behavioral laboratory to complete the EPM test (see below). Directly after the behavioral testing, participants were again seated in the room mentioned above to rate their subjective anxiety level for the different positions on the EPM (center, closed arms, open arms) on a single-item scale from 0 (no anxiety) to 9 (very strong anxiety)^42^.

Most participants were female (n = 107, 67.7%), they were aged between 18 and 50 years (Mean = 25.62 years), born in Germany (n = 154, 86.5%), neither married (n = 9, 5.1%) nor divorced (n = 1, 0.6%), and indicated that they were studying (n = 101, 56.7%) or working (n = 47, 26.4%). Of the female participants, 42% (n = 47) indicated using oral contraceptives. Contraceptive use was assessed but not further analyzed in the present study as a recent study of our lab^47^, that was published after data were collected, could show that even high doses of estrogen do not affect anxiety-related behavior on the human EPM.

### Human elevated plus-maze

All subjects were tested on the human EPM between 3 and 8 pm. The EPM consists of a physical life-sized wooden platform, and its representation is matched in position, distance, and material to a virtual cross in a virtual environment. The cross has four arms of equal length (length 175 cm, width 30 cm), is 20 cm elevated from the floor, and is placed in an indoor experimental room (550 × 550 cm). Participants were guided into the room by an experimenter with their eyes closed. They were instructed to open their eyes only after being fitted with a head-mounted display (HTC Vive^®^, Seattle, USA), which blocked them from seeing the experimental room, and noise-cancelling headphones. The virtual reality environment (A + cross^®^, VirtualRealWorlds.com, Germany) was then started, and participants found themselves in a 550 × 550-cm virtual room in first-person view facing a virtual wooden platform (the maze) in the shape of a cross (350 × 350 cm) on the ground in front of them. A pre-recorded voice instructed participants to step onto the platform and move to the center of the platform, where they were to wait until the environment changed, after which they were allowed to explore the environment while remaining on the platform. The waiting period lasted 90 s. At the end of the waiting period, the behavioral experiment started, and participants no longer found themselves in the virtual room but on a rocky cliff over the sea, still standing in the center of the maze and facing one of the open arms. At this point, additional wind simulation was created using two fans. In this new environment, the center of the maze and two arms (closed arms) were supported by a rocky terrain, while the other two arms (open arms) were suspended over a deep abyss. Participants were permitted to explore the maze for 300 s freely. At the end of the task, headsets were removed while in the experimental room. Exemplary videos can be found in Biedermann et al.^43^.

### Data recording

Data recording was performed through the virtual reality tracking system (HTC Vive Base Station^®^, Seattle, USA) and custom software (A + cross^®^) as described before^42^ using the following parameters: total time spent on open arms (time on open arms), number of entries to open arms, latency for the first entry of an open arm (latency 1st visit) and time until subjects reach the end of an open arm (latency end exploration).

### Brief questionnaire of psychopathic personality traits (FPP)

The Brief Questionnaire of Psychopathic Personality Traits^46^ is a short self-report inventory based on the Psychopathic Personality Inventory^10^ for the measurement of psychopathic personality traits. It consists of 30 items with brief and easy wording and comprises the six scales: *Lack of Empathy*, *Fearlessness*, *Narcissistic Egocentrism*, *Impulsivity*, *Social Manipulation*, and *Power*. Results supported good psychometric properties and high validity of the FPP. The authors define the FPP subscale *Fearlessness* as: “People high in fearlessness have little ability to anticipate negative consequences of their own behavior and are highly insensitive to them.” Item examples are “When doing something, I rarely think that it might go wrong” or “There are few things that frighten me.”

### Sensation seeking scale V (SSSV)

The Zuckerman Sensation Seeking Scale V (SSSV)^44^ consists of 40 forced-choice items and can assess a general measure of sensation seeking by applying a sum score. In addition, it can be split into four factors, each consisting of 10 items: (1) Thrill and Adventure Seeking (TAS; e.g., parachute jumping), (2) Experience Seeking (ES; e.g., exploring strange cities or towns alone), (3) Disinhibition (DIS; e.g., desiring varied sexual experiences), and (4) Boredom Susceptibility (e.g., preference for unpredictable friends).

### Acrophobia questionnaire (AQ)

The Acrophobia Questionnaire^45^ is a well-established self-report tool consisting of 40 Likert-type items regarding anxiety (AQ-anxiety) and avoidance (AQ-avoidance) in 20 height-relevant situations. The psychometric properties are considered good, and it is widely used in anxiety research.

### Statistical analyses

Statistical analyses were carried out using IBM SPSS Statistics 23.0 (IBM Corp., Armonk, NY, USA) and R software (R version 4.0.2). Pearson correlation was performed to assess associations between psychopathic traits and measures of anxiety-related constructs. We moreover divided all participants according to their results on the FPP using quartile split in low, medium, and high FPP and presented descriptive analyses for these three groups^48^.

Thereafter, a multivariate linear regression model was calculated to disentangle the influence of the single subscores of the FPP as independent variables on the four behavioral outcomes as dependent variables. Wilk’s Lambda was used as the multivariate test. In addition to the multivariate model, separate multiple regression models were fitted with the four behavioural outcomes time on open arms, latency open arm exploration, number of entries open arm, and latency open arm end exploration, as well as subjective anxiety as the outcome variables.

All data are given as mean ± standard error (SEM). Statistical significance was set at *p* < 0.05.

## Results

**Figure 1:**
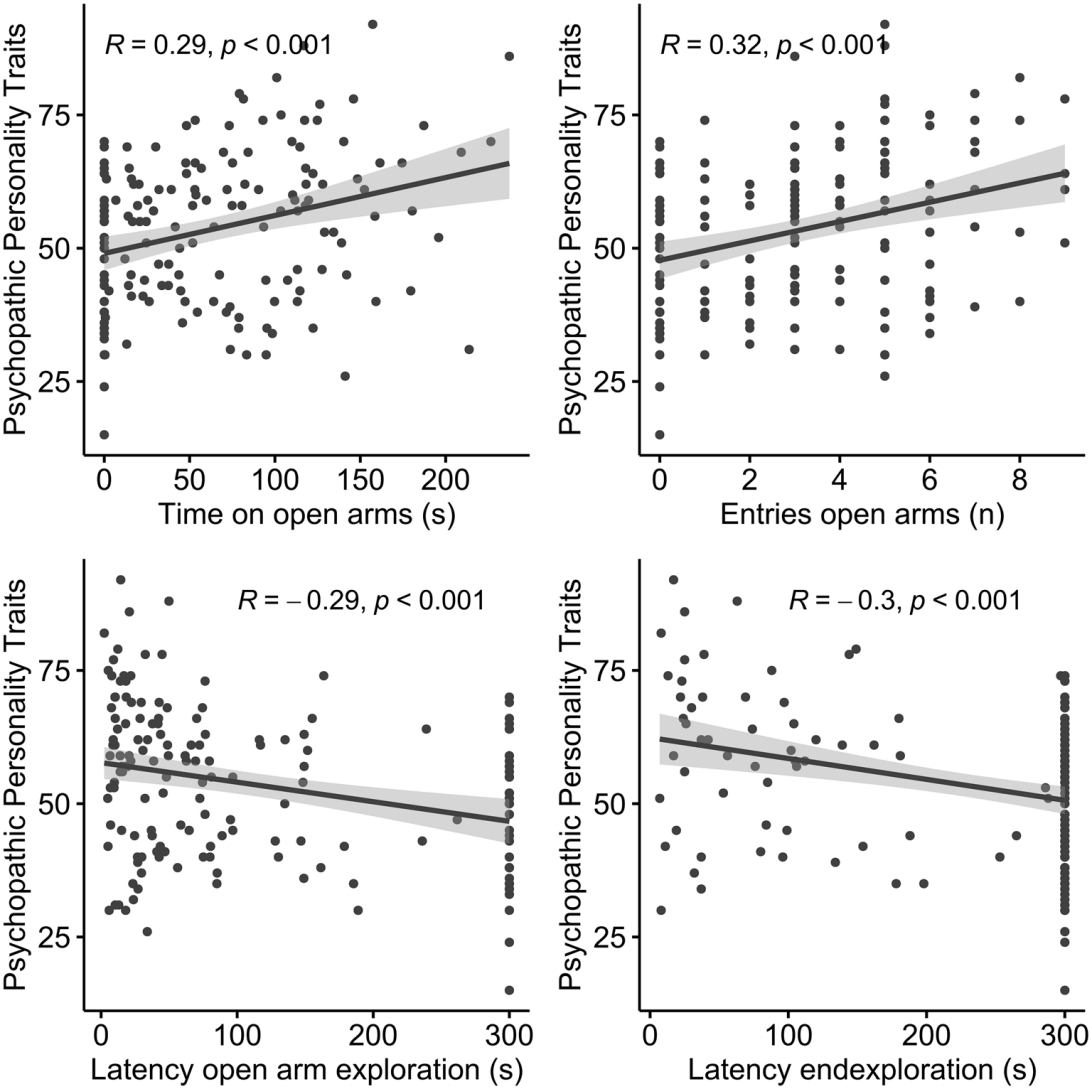
The relation between the core features of anxiety-related behavior on the EPM and sum-scores on the FPP.

Higher psychopathic traits were associated with higher levels of approach and reduced levels of avoidance behavior. In our findings, the FPP sum score correlated significantly with all measures of anxiety-like behavior on the EPM (time on open arms: r = 0.30, *p* < 0.001; number of entries to open arms: r = 0.32, *p* < 0.001; latency open arm exploration: r = − 0.29, *p* < 0.001; latency open arm end exploration: r = − 0.30, *p* < 0.001; Fig. 1). This correlation was still present when controlling for age and gender of participants in a partial correlation (time on open arms: r = 0.26, *p* = 0.002; number of entries to open arms: r = 0.26, *p* = 0.002; latency open arm exploration: r = − 0.23, *p* = 0.006; latency open arm end exploration: r = − 0.21, *p* = 0.012). Interestingly, on the subscale level, not only the subscale *Fearlessness* of the FPP was related to behavior on the EPM but also the other subscales (Table 1).

**Table 1:** Correlations between psychopathic traits and behavioral measures on the EPM as well as subjective anxiety.

		Lack of empathy	Fearlessness	Narcissistic egocentricity	Impulsivity	Social manipulation	Power	FPP sumscore
Latency 1st visit (s)	Pearson correlation	− 0.198*	− 0.359**	− 0.138	− 0.106	− 0.164*	− 0.142	− 0.288**
	Sig. (2-tailed)	0.012	0.000	0.083	0.182	0.039	0.073	0.000
Latency endexploration (s)	Pearson correlation	− 0.124	− 0.221**	− 0.157*	− 0.194*	− 0.175*	− 0.205**	− 0.302**
	Sig. (2-tailed)	0.118	0.005	0.048	0.014	0.027	0.009	0.000
Time on open arms (s)	Pearson correlation	0.175*	0.336**	0.169*	0.155	0.151	0.184*	0.295**
	Sig. (2-tailed)	0.027	0.000	0.033	0.050	0.056	0.020	0.000
Number of entries open arm	Pearson correlation	0.297**	0.299**	0.182*	0.104	0.141	0.174*	0.315**
	Sig. (2-tailed)	0.000	0.000	0.021	0.189	0.076	0.028	0.000
Subjective anxiety	Pearson correlation	− 0.125	− 0.237**	− 0.082	− 0.068	− 0.160*	− 0.087	− 0.226**
	Sig. (2-tailed)	0.115	0.003	0.303	0.390	0.044	0.274	0.004

*. Correlation is significant at the 0.05 level (2-tailed).

**. Correlation is significant at the 0.01 level (2-tailed).

**Figure 2:**
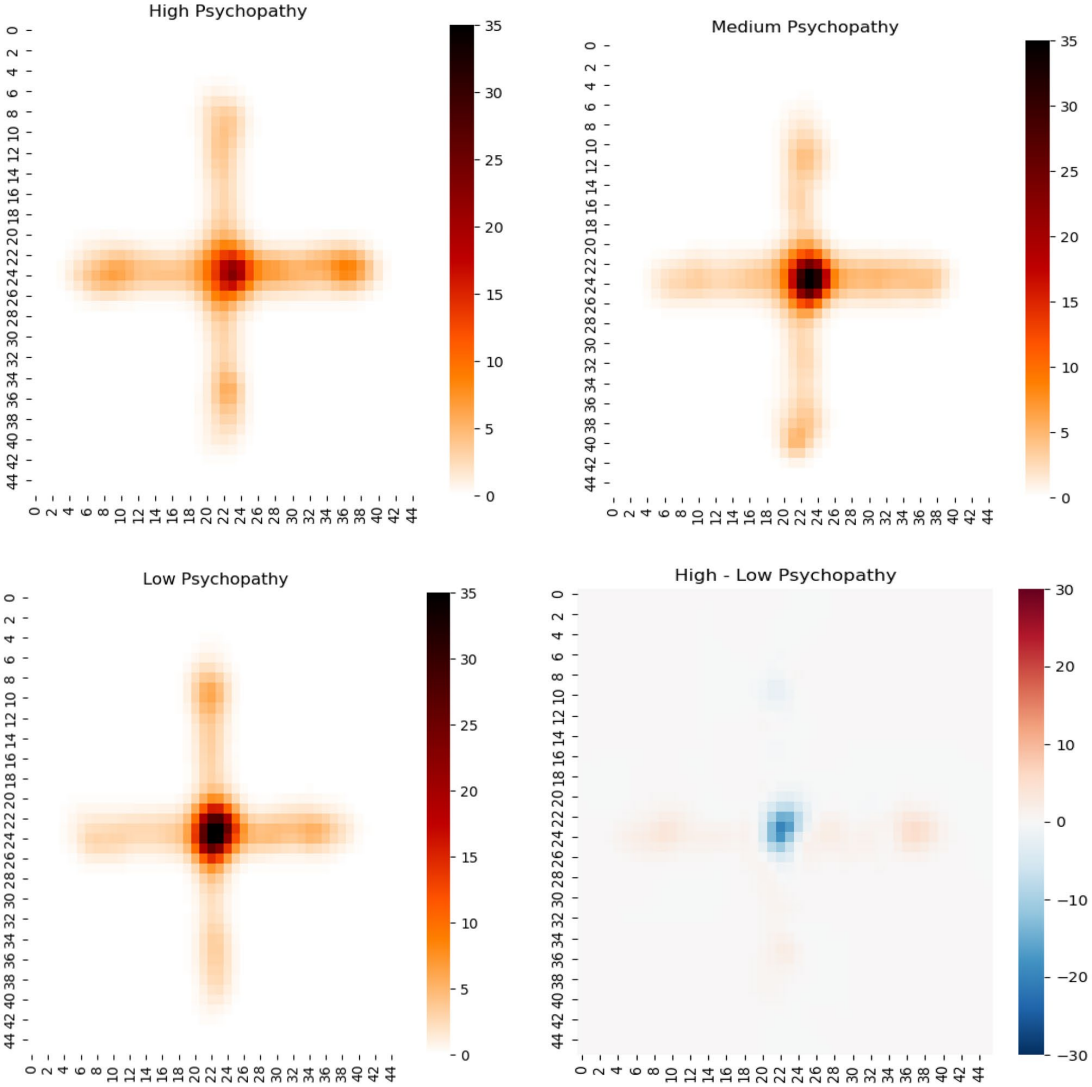
Heatmaps reveal time spent on the EPM in relation to the FPP sum score. The three groups of low, medium and high psychopathy were constructed using a quartile split into low, medium and high FPP. Sociodemographic characteristics of the groups were Low FPP: 74.5% female, 25.5% male, age: Mean = 26.1 years, SD = 5.1 years; Medium FPP: 73.3% female, 26.7 male, age: Mean = 25.4 years, SD = 5.8 years; High FPP: 60.0% female, 40.0 male, age: Mean = 24.6 years, SD = 5.6 years.

Psychopathic traits were also negatively correlated with subjective levels of anxiety on the EPM (r = − 0.23, *p* = 0.004). We also correlated sensation seeking and acrophobia with psychopathic traits as we hypothesized that both measures could be influenced by psychopathic traits. Indeed, sensation seeking (SSSV) (r = 0.33, *p* < 0.001) but not general levels of acrophobia (AQ) (r = − 0.13, *p* = 0.11) was associated with psychopathic traits. Linear regression revealed that the factor *Fearlessness* (*p* = 0.001) on the FPP predicted time on open arms. Latency open-arm exploration was predicted by *Lack of empathy* (*p* = 0.048) and *Fearlessness* (*p* < 0.001) on the FPP. Latency open arm end exploration was predicted by *Impulsivity* (*p* = 0.049) of the FPP. Meanwhile, the number of entries open arm was predicted by *Lack of empathy* (*p* < 0.001) and *Fearlessness* (*p* = 0.012) of the FPP (Table 2). Multivariate analyses revealed that the two subscales *Fearlessness* and *Lack of empathy* were jointly contributing to all models (Table 2). Heatmaps depicting the time participants spent on the EPM in relation to the FPP sum score are presented in Fig. 2.

**Table 2:** Multivariable linear regression models for the association between psychopathic personality traits and elevated plus-maze/subjective anxiety.

Predictor	Outcome variable	Multivariate model (*p*-values)	Time on open arms	Latency open arm exploration	Latency open arm end exploration	Number of entries open arm	Subjective anxiety (not included in the multivariate model)
Lack of empathy		0.029	0.14 (*p* = 0.104)	− 0.16 (*p* = 0.048)	− 0.12 (*p* = 0.153)	0.27 (*p* = 0.001)	0.02 (*p* = 0.839)
Fearlessness		0.004	0.28 (*p* = 0.001)	− 0.31 (*p* < 0.001)	− 0.14 (*p* = 0.110)	0.21 (*p* = 0.012)	− 0.21 (*p* = 0.016)
Narcissistic egocentrism		0.987	− 0.02 (*p* = 0.863)	0.04 (*p* = 0.648)	0.04 (*p* = 0.729)	− 0.01 (*p* = 0.892)	0.09 (*p* = 0.379)
Impulsivity		0.374	0.14 (*p* = 0.110)	− 0.11 (*p* = 0.197)	− 0.17 (*p* = 0.049)	0.11 (*p* = 0.185)	0.08 (*p* = 0.362)
Social manipulation		0.831	0.04 (*p* = 0.696)	− 0.09 (*p* = 0.352)	− 0.09 (*p* = 0.364)	0.06 (*p* = 0.520)	− 0.09 (*p* = 0.340)
Power		0.912	0.00 (*p* = 0.929)	0.05 (*p* = 0.643)	− 0.05 (*p* = 0.649)	0.00 (*p* = 0.999)	− 0.05 (*p* = 0.636)
Model fit: R^2^			0.14	0.16	0.10	0.16	0.07
Sample size		160	160	160	160	160	160

We report standardized regression coefficients with the associated *p*-values.

## Discussion

Our results demonstrate the influence of specific psychopathic personality traits on human behavior in a mixed reality environment. To the best of our knowledge, our study is the first to examine the interplay between psychopathic traits and anxiety, not only on a subjective level but also on a behavioral level. As hypothesized, in our non-clinical sample, a higher sum score of psychopathy correlated with less anxiety-related behavior and lower subjective levels of anxiety. More specifically, our results show an association between the specific subscales of *Fearlessness, Lack of Empathy,* and *Impulsivity* as measured by the FPP, and anxious behavior in the EPM. This is consistent with the findings of Andersen et al. (2021)^15^ and with the often-cited lack of anxiety in psychopathy^10–12^. As the FPP is a derivate of the PPI^10^, it is relevant to note that the subscale *Fearlessness* specifically includes sensation-seeking and physical risk-taking behaviors at the item level. The subscale *Impulsivity*, which has a strong association with anxiety-related behavior, differs from previous findings indicating a positive correlation between Factor 2 psychopathy (antisocial-lifestyle) and anxiety^19^.

In addition, participants in our study who scored higher on psychopathic traits felt significantly less anxious during the experiment on a subjective level, further supporting the lack of anxiety hypothesis and possible deficits in the ability to inhibit potentially inappropriate approach behavior^49,50^. As expected, sensation-seeking correlated positively with psychopathy and with EPM behavioral measures, thus, supporting the idea that risk-taking behavior is associated with psychopathic traits^51^.

In light of the ongoing discussion, our findings cannot infer a general lack of anxiety in psychopathy. Instead, we present evidence for the influence of psychopathic personality traits on specific approach-avoidance behavior as measured by the EPM. Psychopathy is a vital personality construct in clinical and forensic psychology and psychiatry because of its well-established influence on antisocial and delinquent behavior. Our findings raise the question of how far the interplay of psychopathy and anxiety-related behavior influences explicitly criminal behavior. The literature on antisocial behavior and anxiety can be considered mixed^22^. On the one hand, higher levels of anxiety and depression have been found in male offenders with a less severe course of chronic offending compared to men with lifelong persistent antisocial behavior^52^, suggesting a protective effect of anxiety on criminal behavior^53^. On the other hand, several studies describe a relatively strong relationship between anxiety, depression, and antisocial behavior^54–56^.

As expected, the subscale *Fearlessness* significantly influenced nearly every anxiety-related behavior measurement of the EPM, and, in combination with the subscale *Lack of empathy*, remained significant in the multivariate model. The EPM is an established test to detect specifically anxiety-related behavior, and fear and anxiety are considered mainly distinct concepts. Still, fear and anxiety remain closely related adaptive emotions, and due to this overlap between both constructs, our results seem sensible^57–59^.

*Impulsivity* also influenced approach-avoidance behavior in the regression model (latency open arm endexploration), although in the multivariate model it did not contribute to all models. Still, this is interesting, given that personality disorders in ICD-11 will include *Disinhibition* as one of the five trait domain specifiers, including impulsive behavior, besides the trait domain *Dissociality*^60^. Further, high impulsivity is considered to be a significant risk factor for aggressive behavior in general offending and forensic populations^61,62^. While the International Society for Research on Impulsivity defines impulsivity as “behavior without adequate thought, the tendency to act with less forethought than do most individuals of equal ability and knowledge, or a predisposition toward rapid, unplanned reactions to internal or external stimuli without regard to negative consequences of these reactions”^63^, there is, to date, no unitary conceptualization of impulsivity^64,65^. Following the definition above, it appears coherent that impulsive personality traits may be associated with anxiety-related behavior.

The *Lack of empathy* subscale significantly influenced the number of entries on open arms and the latency until participants made the first step on the open arm (latency open arm exploration). It seems reasonable that participants with a specific lack of empathy may also be more likely to act against their natural protective instincts or protective impulses in a usually anxiety-inducing situation. With our findings that specifically the subscales *Fearlessness and Lack of empathy are jointly contributing to the prediction* of anxious behavior in the EPM, our results are consistent with the approach of diagnosing personality disorders at the domain level in ICD-11.

It seems understandable that a tendency to engage in approach behavior in an anxiety-provoking environment could potentially lead to problematic, risky, and even criminal behavior. To broaden our knowledge in this area, it would be of great interest to conduct a further EPM study with a sample of criminal offenders in a prison setting to test whether our findings can be replicated in a population with higher levels of psychopathy.

One of the strengths of our study is the innovative study design addressing actual behavior and not just surrogates of behavior on a questionnaire level or computer task, which makes it possible to show the potential influence of psychopathic traits on anxiety-related behavior. A limitation of our study is the use of a healthy study population and, thus, lacking individuals at the extreme ends of the assessed dimensions, e.g., testing an offender sample with higher psychopathy scores. Further, evaluating psychopathy through a self-report instrument can be viewed as critical, the gold standard in psychopathy research being the *Psychopathy Checklist-Revised*^1^, a semi-structured interview by trained specialists. In our study, we solely measured the actual behavior and no additional physiological reactions, such as heart rate, skin conductance levels, or endocrinological parameters (e.g., cortisol), which could lead to broader insights in relation to psychopathic traits.

In conclusion, by using an EPM in mixed reality we here show for the first time that actual anxiety-related behavior is correlated with psychopathic traits in healthy individuals. We identified that particularly the subscales *Fearlessness* and *Lack of empathy* were related to anxiety-related behaviors (Supplementary file).

### Supplementary Information

Supplementary Tables.

## Data Availability

All data generated or analyzed during this study are included in this published article and its additional files.
