# Supplementary material for: The impact of psychopathic traits on anxiety-related behaviors in a mixed reality environment
Source: Sci Rep. 2024 May 23;14:11832. doi: 10.1038/s41598-024-62438-9 (PMC11116403; doi:10.1038/s41598-024-62438-9)
Supplement: Supplementary file 1 — Supplementary Tables. [file 41598_2024_62438_MOESM1_ESM.pdf]

The impact of psychopathic traits on anxiety-related behaviors in a mixed reality environment

Alexander Voulgaris[1]*, Sarah V. Biedermann[2], Daniel Biedermann[2,3], Susanne Bründl[3], Lateefah Roth[3], Christian Wiessner[4], Peer Briken[1], Johannes Fuss[3]*

Supplementary Table 1: Pearson correlations between psychopathic traits, behavioral measures on the EPM, subjective anxiety, acrophobia and sensation seeking

	Lack of empathy	Fearlessness	Narcissistic Egocentricity	Impulsivity	Social Manipulation	Power	Latency 1st visit (s)	Latency endexploration (s)	Time on open arms (s)	Number of entries open arm	Subjective anxiety	Acrophobia	Sensation seeking
Lack of empathy	1	0.235**	0.266**	-0.148	0.001	0.139	-0.198*	-0.124	0.175*	0.297**	-0.060	-0.091	0.203*
Fearlessness		1	0.303**	0.151*	0.326**	0.347**	-0.359**	-0.221**	0.336**	0.299**	-0.232**	-0.283**	0.325**
Narcissistic Egocentricity			1	0.269**	0.437**	0.577**	-0.138	-0.157*	0.169*	0.182*	-0.007	-0.032	0.146
Impulsivity				1	0.153*	0.364**	-0.106	-0.194*	0.155	0.104	0.042	0.150	0.107
Social Manipulation					1	0.564**	-0.164*	-0.175*	0.151	0.141	-0.097	-0.147	0.241**
Power						1	-0.142	-0.205**	0.184*	0.174*	-0.056	0.023	0.141
Latency 1st visit (s)							1	0.540**	-0.704**	-0.746**	0.563**	0.430**	-0.280**
Latency endexploration (s)								1	-0.645**	-0.451**	0.325**	0.194*	-0.114
Time on open arms (s)									1	0.633**	-0.469**	-0.323**	0.276**
Number of entries open arm										1	-0.397**	-0.299**	0.312**
Subjective anxiety											1	0.460**	-0.201*
Acrophobia												1	-0.290**
Sensation seeking													1

*: Correlation is significant at the 0.05 level (2-tailed)
**: Correlation is significant at the 0.01 level (2-tailed)
